# Early progressive mobility to improve neurodevelopment of infants with severe bronchopulmonary dysplasia at a level IV neonatal intensive care unit: a prospective cohort study

**DOI:** 10.1038/s41372-024-02168-y

**Published:** 2024-11-13

**Authors:** Heidi Morris, Kathleen Nilan, Meghan Burkhardt, Audrey Wood, Molly Passarella, Kathleen Gibbs, Sara B. DeMauro

**Affiliations:** 1 Division of Neonatology, Children’s Hospital of Philadelphia, Philadelphia, PA USA; 2 Newborn and Infant Chronic Lung Disease Program, Children’s Hospital of Philadelphia, Philadelphia, PA USA; 3 Occupational Therapy Department, Children’s Hospital of Philadelphia, Philadelphia, PA USA; 4 Physical Therapy Department, Children’s Hospital of Philadelphia, Philadelphia, PA USA; 5 Neonatal Follow-up Program, Children’s Hospital of Philadelphia, Philadelphia, PA USA; 6 Center for Perinatal and Pediatric Health Disparities Research, Children’s Hospital of Philadelphia, Philadelphia, PA USA; 7 University of Pennsylvania Perelman School of Medicine, Philadelphia, PA USA

**Keywords:** Paediatrics, Outcomes research

## Abstract

**Objective:**

To measure the feasibility of early progressive mobility (EPM) in intubated infants with severe bronchopulmonary dysplasia (BPD) and compare neurodevelopmental skill acquisition of these infants before and after implementation of a clinical EPM program.

**Study design:**

Single-center pre-post intervention prospective cohort study in a level IV Neonatal Intensive Care Unit (NICU) from 2019–2022. Bivariate tests compared EPM interventions and results of serial Test of Infant Motor Performance (TIMP) assessments in 32 intubated infants with severe BPD cared for during two epochs, before and after NICU-wide EPM implementation.

**Results:**

Infants in epoch 2 experienced significantly more EPM interventions than infants in epoch 1. Infants in epoch 2 also had more advanced motor skills on the TIMP than infants in epoch 1. There were no unplanned extubations.

**Conclusions:**

We demonstrated successful implementation of EPM in this high-risk population with evidence of beneficial impacts on early motor development.

## Introduction

Infants with bronchopulmonary dysplasia (BPD) are at high risk for adverse medical and developmental sequelae starting in infancy and progressing through childhood [1]. Infants with grade 3 BPD, who are intubated until at least 36 weeks postmenstrual age (PMA), spend prolonged periods of time laying supine rather than participating in age-appropriate positioning and developmental activities, potentially limiting skill acquisition during a critical neurodevelopmental period [2, 3]. We have previously demonstrated both failure to acquire typical early motor developmental milestones, including reaching, rolling, and sitting, and loss of milestones among infants who require prolonged respiratory support for management of severe BPD [4]. Later in life, infants and young children with BPD have continued delays in motor skills which are associated with delays in other domains of development, such as cognition and language [2].

The Newborn/Infant Chronic Lung Disease Program (NeoCLD) at the Children’s Hospital of Philadelphia (CHOP) is a referral-based program within the newborn/infant intensive care unit (NICU) that provides multidisciplinary care for neonates and infants with severe lung disease. These patients require critical and specialized care that often involves high levels of respiratory support, including invasive mechanical ventilation, for several months. Due to the infants’ critical illness, concern for disruption of medical devices including endotracheal tubes, and often poor tolerance to position changes, these infants have traditionally spent most of their time in the supine position.

Early progressive mobility (EPM), the practice of moving critically ill patients to sitting and out-of-bed positions in a stepwise fashion, has been studied in adult and pediatric intensive care units. In these older patients, it is well-established that utilizing EPM early in illness reduces morbidity and mortality [5, 6]. Early mobilization has also been shown to be safe, without adverse events reported in the existing studies of intubated pediatric patients [7, 8]. To date, EPM has not been studied in critically ill infants who require prolonged and invasive respiratory support.

The objectives of the current study were to evaluate the feasibility and safety of EPM in intubated NICU patients with severe BPD and to characterize short-term neurodevelopmental skill acquisition in this population following the implementation of a unit-wide clinical EPM program.

## Methods

### Study design, setting & participants

We performed a single-center pre-post intervention prospective cohort study and followed STROBE reporting guidelines [9]. Beginning in April 2020, a unit-wide clinical EPM program was launched in CHOP’s level-IV NICU. Implementation of the program was multifaceted and included development of a holding and mobility job aid inclusive of nurses, physical and occupational therapists, and caregivers; a mandatory electronic learning module for nurses, frontline clinicians, and physical and occupational therapists; supplemental bedside education of nursing staff by physical and occupational therapists; a hospital-wide standard therapy order; multiple educational reinforcement techniques via signage and visual reminders; and a rounding tool in which a question about the infants’ out of bed experiences was answered by the medical providers each Friday during clinical work rounds. Therapy is routinely ordered in the electronic medical record for all patients who are developmentally and clinically appropriate for movement as evidenced by ability to maintain state control and hemodynamic stability, and who do not fall under specific exclusion criteria (for example dependence on a high frequency ventilator or immediately post operative).

For this study, we enrolled 32 intubated infants with severe BPD who were managed by the NeoCLD Program between April 2019 and January 2022. The NeoCLD program is a multidisciplinary regional referral service specializing in the management of infants with severe BPD. Study participants were enrolled during two epochs: pre- and post-EPM implementation. Infants in epoch 1 were enrolled and completed study activities between April 2019 and April 2020 and infants in epoch 2 were enrolled between June 2020 and January 2022. We initially planned for 20 participants in each epoch, however therapy staffing was unexpectedly limited by hospital mandate in the setting of the COVID-19 pandemic. Thus, 16 subjects were enrolled in the pre-EPM implementation epoch; this number was then matched in the post-EPM implementation epoch. Eligible infants had a diagnosis of severe BPD, had a post menstrual age (PMA) of 40 weeks or greater, and were intubated with an endotracheal tube (ETT) at the time of enrollment. We excluded infants with diagnosis of critical airway, infants who were pharmacologically muscle relaxed, and infants for whom therapy was not ordered because they were deemed medically unstable by their clinical providers.

### Study variables & measures

Clinical and demographic data were extracted from the electronic medical record. Baseline demographic data were infant sex, gestational age (GA) in completed weeks at birth, and birth length and weight. Additional clinical and outcome variables included: length of NICU and hospital stay, serial growth data, ultimate tracheostomy status, and survival.

A modified version of the Test of Infant Motor Performance (TIMP) was used to characterize short-term motor skill development in the study participants. The TIMP is a validated, standardized test of functional motor skills in infants that can discriminate among young infants with varying risk for motor delays, starting at 40 weeks PMA [10–12]. The TIMP was not developed for use in children who are intubated and our study team determined that most prone items were inappropriate for use in this population. Therefore, a modified version of the TIMP, which includes 13 observed items and 4 elicited items related to head control and is used routinely to assess developmental progress in patients managed by the NeoCLD program, was used as the primary outcome for the current study. Assessments are documented in a physical or occupational therapy progress note in the electronic medical record. For the purposes of this study, the TIMP was administered to each participating infant during routine therapy sessions on two occasions approximately 4–6 weeks apart, depending on therapist availability and infant clinical status, beginning no sooner than 40 weeks PMA.

To evaluate feasibility and demonstrate successful implementation of EPM, granular data on infant movement experiences were collected from the electronic medical record. These data included numbers of physical or occupational therapy sessions; positioning by therapists or nurses in supine, prone, sitting up in bed or in a seating device; skin-to-skin holding with a parent or caregiver; times out of bed (OOB) on the floor/mat; and positioning in tilt and side-lying during the time interval between the 2 TIMPs. Safety data included monitoring for any cases of unplanned extubations in study subjects, exposure to additional sedating medications (any opioid, benzodiazepine, or other medication administered for sedation and/or anxiolysis), and respiratory severity score (product of mean airway pressure and fraction of inspired oxygen) on days of TIMP assessments as indicators of infant tolerance of progressive mobility.

### Statistical analysis

The planned sample size of 20 participants in each group was expected to provide >80% power to detect a difference in 3 points in the change in TIMP over time between the pre-EPM group (epoch 1) and post-EPM group (epoch 2). As described above, the final sample size was smaller due to pandemic-related impacts on therapist presence in our NICU.

The primary analysis is a difference in differences approach to compare the change in performance of the 17 items of the TIMP over 4–6 weeks in epoch 1 as compared to epoch 2. Bivariate comparisons between groups and between time points in each group were performed with Fisher’s exact, Kruskal-Wallis, and t-tests. We used linear regression models to evaluate change in elicited, observed, and total TIMP scores adjusted for baseline or starting values in the two study groups. All analyses were performed with STATA/SE v16.1 (College Station, TX) and *p* < 0.05 was considered significant. The study was deemed exempt by the CHOP Institutional Review Board; therefore, consent was not required.

## Results

There were 32 infants who met eligibility criteria enrolled in this study. Sixteen were enrolled in the pre-EPM implementation group and 16 in the post-EPM implementation group. Basic demographics, including birthweight, sex, and gestational age were similar in the two groups (Table 1). PMA and respiratory severity score were similar between the pre-EPM group (epoch 1) and post-EPM group (epoch 2) at each of the two TIMP timepoints (Table 1). There was an average of 32 and 40 days (*p* = 0.11) between documented TIMP administrations in the pre- and post-EPM implementation groups, respectively.

**Table 1:** Infant characteristics.

	Epoch 1 (Pre-EPM education)	Epoch 2 (Post-EPM education)	p-value
BASELINE	*n* = 16	*n* = 16	
Female – *n* (%)	6 (38%)	8 (50%)	0.72
Birthweight - grams	651 +/− 229	643 +/− 105	0.90
Gestational age - weeks	25.6 +/− 2.1	24.8 +/− 1.7	0.24
FIRST TIMP	*n* = 15	*n* = 16	
Postmenstrual age - weeks	44.8 +/− 3.6	43.4 +/− 3.2	0.29
Respiratory severity score	6.9 +/− 2.3	6.4 +/− 2.8	0.55
Standing sedation – *n* (%)	12 (75%)	16 (100%)	0.10
As needed sedation – *n* (%)	4 (25%)	8 (50%)	0.27
SECOND TIMP	*n* = 15	n = 14	
Postmenstrual age - weeks	49.5 +/− 3.8	49.1 +/− 4.0	0.79
Respiratory severity score	5.6 +/− 2.2	4.4 +/− 3.6	0.29
Standing sedation – *n* (%)	11 (69%)	13 (81%)	0.69
As needed sedation – *n* (%)	11 (73%)	6 (43%)	0.14
CHANGE BETWEEN TIMPs	*n* = 15	*n* = 14	
Days between TIMPs	32 +/− 15	40 +/− 9	0.11
Change in respiratory severity score between TIMPs	−1.1 +/− 1.6	−1.9 +/− 3.1	0.38

*TIMP* Test of infant motor performance.

Physical and occupational therapy sessions were attempted and completed significantly more often during epoch 2 (Table 2). Furthermore, therapists and bedside nurses more frequently changed the infants’ positions, including sitting them up and moving them to a seated position in a chair or other positioning device after implementation of EPM. Caregivers were far more likely to hold infants skin-to-skin during epoch 2. In balance, infants in epoch 2 were less often positioned in a “tilt” position or side-lying in bed compared to infants in epoch 1. There were no documented unplanned extubations during therapy or movement sessions. Exposure to standing or as needed (“PRN”) sedation medications and respiratory severity scores were similar between the two epochs at each of the two time points (Table 1).

**Table 2:** EPM Implementation – numbers of documented interventions per 7 days between TIMP assessments.

	Epoch 1 (Pre-EPM education, *n* = 15)	Epoch 2 (Post-EPM education, *n* = 14)	*p*-value
Physical or occupational therapy (PT/OT) sessions attempted	2.4 +/− 0.6	3.4 +/− 1.1	0.003
PT/OT sessions completed	1.6 +/− 0.5	2.3 +/− 0.8	0.009
Prone position with nurse or PT/OT	1.5 +/− 1.0	2.7 +/− 2.2	0.07
Sitting up with nurse or PT/OT	1.1 +/− 0.5	1.8 +/− 0.6	0.002
Placed in chair with nurse or PT/OT	0.3 +/− 0.6	2.8 +/− 3.0	0.003
Skin to skin	1.8 +/− 1.3	4.3 +/− 2.2	<0.001
Tilted in bed	12.4 +/− 4.9	8.7 +/− 2.7	0.02
Side lying in bed	17.5 +/− 5.1	16.1 +/− 6.1	0.50

Total TIMP raw score was significantly higher during epoch 2 at both the first TIMP assessment timepoint and the second TIMP assessment timepoint (Table 3). Similar patterns were seen in the elicited, but not the observed, TIMP items. Over the 6-week observation study period, total TIMP raw score increased by 2.2 points among infants in epoch 1 compared to 3.1 points among infants in epoch 2 (*p* = 0.36).

**Table 3:** TIMP assessments.

	Epoch 1 (Pre-EPM education)	Epoch 2 (Post-EPM education)	p-value
FIRST TIMP			
Elicited items	2.5 +/− 2.3	4.3 +/− 1.9	0.02
Observed items	6.7 +/− 1.5	6.9 +/− 1.6	0.72
Total raw score	9.2 +/− 2.7	11.2 +/− 2.9	0.06
SECOND TIMP			
Elicited items	3.7 +/− 1.5	6.4 +/− 2.2	<0.001
Observed items	7.7 +/− 0.5	8.1 +/− 1.0	0.12
Total raw score	11.4 +/− 1.5	14.5 +/− 2.8	<0.001
CHANGE IN TIMP			
Elicited items	1.3 +/− 2.4	2.1 +/− 2.7	0.36
Observed items	0.9 +/− 1.4	1.0 +/− 1.5	0.91
Total raw score	2.2 +/− 2.8	3.1 +/− 2.7	0.36

We used linear regression models adjusted for baseline value of the TIMP to calculate predicted change in elicited, observed, and total raw TIMP scores between the two observation timepoints (Table 4). Modest increases in observed and total scores were predicted for infants enrolled during epoch 1. In contrast, elicited and total scores were predicted to increase significantly for infants enrolled during epoch 2. After implementation of EPM, predicted average increase in total TIMP raw score over the 4–6 week observation period was 3.9 points, compared to 1.5 points before implementation of EPM.

**Table 4:** Change in TIMP Scores.

Predicted change in TIMP	Pre-EPM	Post-EPM
Elicited items	0.6 (−0.5–1.6)	2.9 (1.8–4.0)
Observed items	0.8 (0.4–1.2)	1.2 (0.8–1.6)
Total score	1.5 (0.4–2.6)	3.9 (2.7–5.0)

## Discussion

We demonstrate successful implementation of an EPM program in critically ill intubated infants with severe BPD in a level IV neonatal intensive care unit (NICU). We achieved this without experiencing unplanned extubations during EPM. In addition, study subjects did not experience sustained increases in respiratory support or significant additional exposure to sedating medications associated with participating in EPM. Our data suggest that infants exposed to EPM may have improved developmental skill acquisition, potentially beginning before term corrected age. Our findings were similar to previous studies in intubated pediatric patients that demonstrated early mobilization to be safe and overall well tolerated [5, 6].

In most NICUs, the current culture and environment may not support the necessary movement and routine out of bed experiences necessary to promote motor skill acquisition in intubated and critically ill infants and neonates [13]. Out of concern for safety – namely in an effort to avoid unplanned extubations – bedside providers may be hesitant to adopt a culture of mobility. However, by establishing specific guidelines and providing targeted education about the potential benefits of EPM, we found that the practice was widely accepted by nurses, therapists, and caregivers. EPM was readily integrated as the standard of care in our NICU, as evidenced by infants in the second epoch experiencing significantly more therapy sessions, position changes, and transfers out of bed. We also found that infants in the second epoch were less often positioned in “tilt,” suggesting that the bedside providers were selecting sitting or prone positions in place of tilted positions for their patients. Consistent with studies in the pediatric intensive care literature, we did not identify any safety events during EPM in our intubated infants [14].

In critically ill older pediatric and adult populations, the goal of EPM is to return patients to the state of health they were in prior to an acute illness. In contrast, the goal of EPM in this infant population is to facilitate development of age-appropriate motor skills and ultimately prevent longer-term developmental delays. When compared to the pre-EPM epoch, our post-EPM implementation group started with higher TIMP scores and we found significantly greater increases in all portions of the TIMP throughout the observation period. We hypothesize that the higher TIMP starting value during epoch 2 represents implementation of EPM starting well before 40 weeks PMA. Out of an abundance of caution for the fragility of pre-term infants, we did not administer the TIMP at earlier time points in the current study. Future work to further delineate the impacts of EPM on motor skill acquisition before full-term corrected age will be an important next step in determining how and when to implement EPM in routine neonatal clinical care.

The development of postural control begins prenatally as the fetus moves within the uterine environment and continues after birth as an infant learns to overcome the forces of gravity. The acquisition of head control is one of the first motor skills to be mastered by young infants, emerging at about one month corrected age. Like other areas of motor development, development of head control requires experience and frequent opportunities for practice [6–8]. Practice is achieved through daily routines with caregivers such as dressing, feeding, bathing, and being carried from place to place [8, 10–12]. Enhanced movement experiences through therapy improve the development of head control in healthy infants and decreased experience using postural control strategies during early infancy is associated with delayed acquisition of head control [15]. Early skills such as head control lay the foundation for subsequent motor skills as well as the development of skills in other domains such as vision, cognitive, social interaction, and feeding skills [16]. Given difficulties in stabilizing the airway and concern for endotracheal tube dislodgement, extremely preterm infants with BPD have traditionally been afforded fewer opportunities to experience upright positions and develop head righting and control than non-hospitalized infants or even hospitalized infants who do not require invasive respiratory support. Thus, the faster acquisition of elicited head control milestones observed in infants exposed to EPM in the current study may prepare our patients for more timely development of more complex skills across a variety of important neurodevelopmental domains.

This study demonstrates that the practice of EPM can be successfully and safely implemented in a critically ill and high-risk infant population. Furthermore, our study suggests that EPM may provide benefits by supporting acquisition of early motor milestones. However, this study is not without limitations. Although we began collecting data on infants at 40 weeks PMA, many infants in the second epoch started EPM in the weeks prior, potentially impacting observed TIMP scores. The COVID pandemic may have impacted the exposures and outcomes measured in the current study, and all therapy and feasibility data were obtained from the medical record. Additionally, while we hypothesize that early motor skill acquisition translates into sustained improvements in motor development, we do not yet have any longer-term evidence of such benefits.

Our novel approach to EPM in a NICU with the ultimate goal of improving long-term neurodevelopment in critically ill infants could be further advanced with larger studies and with the addition of post-NICU follow up and longer-term data collection. In order to make such studies possible, we hope that other institutions will adapt their own NICU EPM programs. In order to implement a successful program we recommend having detailed, unit-specific EPM guidelines developed by a multidisciplinary team, inclusive of PT/OT, nursing, clinicians, and caregivers, and developing universal and consistent EPM education for staff.

## Data Availability

The data that support the findings of this study are available from the corresponding author upon reasonable request.
